# Effects of early pregnancy on NOD-like receptor expression in the ovine endometrium

**DOI:** 10.3389/fvets.2024.1384386

**Published:** 2024-06-05

**Authors:** Leying Zhang, Jiabao Cai, Xinxin Wang, Zhen Yang, Haiquan Ding, Ling Yang

**Affiliations:** School of Life Sciences and Food Engineering, Hebei University of Engineering, Handan, China

**Keywords:** endometrium, immunology, NOD-like receptor, pregnancy, sheep

## Abstract

**Introduction:**

Nucleotide-binding domain (NOD)-like receptors (NLRs) are expressed in the endometrium, and involved in modulating the female innate immune responses. There are conceptus-endometrial interactions during pregnancy, which ensure immune homeostasis of the maternal-fetal interface. The purpose of this study was to explore the effects of early pregnancy on NLR expression in the ovine endometrium.

**Methods:**

Endometrial tissues were collected at day 16 of the estrous cycle, and at days 13, 16 and 25 of pregnancy (*n* = 6 for each group), and RT-qPCR, western blot and immunohistochemistry analysis were used to analyze the expression of NLRs, including NOD1, NOD2, major histocompatibility complex class II transactivator (CIITA), neuronal apoptosis inhibitor protein (NAIP), NLR family, pyrin domain-containing 1 (NLRP1), NLRP3 and NLRP7.

**Results:**

Expression levels of NOD1, NOD2, NAIP, CIITA, NLRP1 and NLRP3 declined, but expression level of NLRP7 increased in the endometria during early pregnancy compared with nonpregnant ewes. In addition, NOD2 and CIITA proteins were located in the endometrium in a protein type-, cell type- and pregnancy status-specific manner.

**Discussion:**

Early pregnancy modulated expression of NLR family in the ovine endometrium, which may be essential for conceptus-endometrial interactions and maternal-fetal interface immune homeostasis.

## 1 Introduction

During pregnancy, the placenta interacts with the maternal immune system, which does not evoke the robust cellular and humoral immune responses from the mother (1). The developmental conceptus expresses paternal antigens, which crosstalk with the maternal immune system, and creates a maternal–fetal interface immune homeostasis. The endometria are modulated by maternal endocrine and immune systems to prepare for implantation and pregnancy maintenance (2). Pregnancy recognition involves specific hormones, such as chorionic gonadotropin in primates, prolactin in rodents, and interferon tau (IFNT) in ruminants, so the mechanism for signaling pregnancy recognition is highly variable among species of mammals (3). IFNT regulates the expression of interferon regulatory factor 2 and the mechanistic target of rapamycin in the endometria to ensure proliferation, migration, and gene expression in the trophectoderm cells (4).

IFNT stimulates the expression of interferon-stimulated genes (ISGs) in endometrial tissues, as well as in extra-uterine tissues or organs, including blood cells, luteal tissue, and immune organs [such as the bone marrow (5), thymus (6), spleen (7), and lymph nodes (8)] during pregnancy, which modulates the uterine environment and maternal immune responses during normal implantation (9). The conceptus avoids maternal immune detection by modulating the immune interaction between the fetus and mother at the interface of placenta and endometrium, which is related to high concentrations of circulating progesterone (P_4_) and conceptus signal (IFNT) in domestic ruminants (10).

Nucleotide-binding oligomerization domain 1 (NOD1) and NOD2 are present in the human endometrium, which play a role in innate immune protection in the uterus (11). The NOD-like receptor (NLR) family mainly includes class II histocompatibility complex (MHC) transactivator (CIITA), neuronal apoptosis inhibitor protein (NAIP), NOD1, NOD2, nucleotide-binding oligomerization domain, leucine-rich repeat, and pyrin domain-containing (NLRP) (12). NOD1 and NOD2 signaling modulate metabolic, autoimmune, and inflammatory diseases, which is mediated by nuclear factor-κB (NF-κB) signaling, interferon mitogen-associated protein kinase signaling, and inflammasome activation (13). Our previous studies have reported that early pregnancy regulated the expression of the NLR family in the spleen, liver, thymus, and lymph nodes of ewes in a tissue-specific manner, which were involved in modulating maternal immune responses (14–17). It was hypothesized that the expression of the NLR family in the ovine endometrium was modified by early pregnancy via IFNT and P_4_, which may be implicated in regulating maternal endometrial immune responses and contributing to pregnancy establishment. This study aimed to analyze the expression of NOD1, NOD2, CIITA, NAIP, NLRP1, NLRP3, and NLRP7 in the endometrium of ewes during early pregnancy, which will be beneficial for exploring the maternal–fetal interface immune homeostasis during early pregnancy.

## 2 Materials and methods

### 2.1 Animals and experimental design

Chinese Small Tail Han ewes with normal estrous cycles and approximately the same age (18 months) were selected for this study. Twenty-four ewes were randomly divided into four groups (*n* = 6 for each group). The ewes were paired with vasectomized rams to determine the day of estrus (day 0) and run with either intact rams (resulting in pregnant ewes) or not (resulting in non-pregnant ewes). All ewes were slaughtered on days 13, 16, and 25 post-breeding or day 16 of the estrous cycle for sampling endometrial tissues after the conceptus had been disconnected, and pregnancy was confirmed by observing one conceptus in the uterus along with one corpus luteum in the ovaries. Progesterone levels in the plasma were significantly higher on days 12–13 and lower on days 15–16 during the ovine estrous cycle (18). IFNT and additional proteins secreted by the trophoblasts of blastocysts in the uterus were detected between days 14 and 21 in sheep (19). Therefore, samples of endometria were obtained from the ewes on days 13, 16, and 25 post-breeding or day 16 of the estrous cycle. Endometrial cross-section pieces (0.5 cm^3^) were fixed in fresh 4% (w/v) paraformaldehyde in phosphate-buffered saline (pH 7.4) for subsequent immunohistochemical analysis, and the remaining portions were frozen in liquid nitrogen and stored at −80°C for following mRNA and protein analyses.

### 2.2 RNA extraction and RT-qPCR assay

Total RNA was extracted from the endometrial samples using the TRIzol reagent (Tiangen Biotech Co., Ltd., Beijing), and a FastQuant RT kit with DNase (Tiangen Biotech) was used to reverse transcribe an equal amount of RNA to cDNA from each sample. A control that contained all reaction components except for the reverse transcriptase was used to test for contaminating DNA. Primer sequences of NLR family genes and *GAPDH* were designed and synthesized by Sangon Biotech Co., Ltd. (Shanghai, China) (Table 1), and real-time PCR was conducted using a SuperReal PreMix Plus kit (Tiangen Biotech) on a Bio-Rad CFX96 real-time PCR system (Bio-Rad Laboratories, Inc., CA, United States) using only H_2_O as no template control. The amplifying conditions for genes were as follows: denaturation at 95°C for 10 min, 40 cycles of denaturation (95°C for 10 s), annealing (59°C to 62°C for 20 s), and extension (72°C for 25 s), followed by final extension (72°C for 7 min). The annealing temperatures were 60.5°C for *NOD1* and *CIITA*, or 62°C for *NOD2*, 59.5°C for *NAIP*, 60°C for *NLRP1*, 59°C for *NLRP3*, and 61°C for *NLRP7*. The amplification efficiencies of the primer sequences were evaluated before quantification and were in an acceptable range (between 0.9 and 1.0). Mean threshold cycle values (Ct) for the target genes and Ct values for the reference gene (*GAPDH*) of each sample were calculated from triplicate wells, and quantitation of the relative levels of mRNA was carried out by the comparative 2^−ΔΔCt^ method (20). Mean CT values from the samples at day 16 of the estrous cycle were used as reference points to determine the relative levels of mRNA transcripts.

**Table 1:** Primers used for RT-qPCR.

Gene	Primer	Sequence	Size (bp)	Accession numbers
*NOD1*	Forward	CCTTGGCTGTCAGAGATTGGCTTC	94	XM_042248630.1
	Reverse	GCTTCTGGCTGTATCTGCTCACTG		
*NOD2*	Forward	TGCCATCCTCGCTCAGACATCTC	117	XM_042231601.1
	Reverse	CAGCCACACTGCCCTCTTTGC		
*CIITA*	Forward	GCACCTCCTTCCAGTTCCTTGTTG	119	XM_042239890.1
	Reverse	CCTGTCCCAGTCCCTGAGATCG		
*NAIP*	Forward	TTGTCCAGCAGTGTCAGCATCTTC	82	XM_012096791.3
	Reverse	ATTTCCACCACGCTGTCATCATCC		
*NLRP1*	Forward	AAGGAGGTGACCGAGATGCTGAG	143	XM_012185551.4
	Reverse	TGCCGCTTGAGTGAGGATGTATTG		
*NLRP3*	Forward	CTCTGGTTGGTCAGTTGCTGTCTC	81	XM_042250402.1
	Reverse	GGTCAGGGAATGGTTGGTGCTTAG		
*NLRP7*	Forward	GCCTGCTACTCGTTCATCCATCTC	90	XM_004015893.5
	Reverse	CCCTTCCTCCTCCTGCTCTTCC		
*GAPDH*	Forward	GGGTCATCATCTCTGCACCT	176	NM_001190390.1
	Reverse	GGTCATAAGTCCCTCCACGA		

### 2.3 Western blot analysis

RIPA lysis buffer containing phenylmethanesulfonyl fluoride was used to prepare the total proteins of the endometrial samples on ice, and the Bradford method was used to detect the protein concentration. SDS-PAGE (5% stacking and 12% separating gels) was used to separate the total proteins (10 μg per well), and the separated proteins were electro-transferred onto the surface of methanol-activated polyvinylidene fluoride membranes (Millipore, Bedford, MA, United States). The membranes were blocked using 5% skim milk and then exposed to primary antibodies in a 1:1,000 dilution at 4°C overnight. The primary antibodies include a mouse anti-NOD1 monoclonal antibody (Santa Cruz Biotechnology, Santa Cruz, CA, United States, sc-398696), a mouse anti-NOD2 monoclonal antibody (Santa Cruz Biotechnology, sc-56168), a mouse anti-CIITA monoclonal antibody (Santa Cruz Biotechnology, sc-13556), a rabbit anti-NAIP polyclonal antibody (Abcam, Cambridge, United Kingdom, ab25968), a mouse anti-NLRP1 monoclonal antibody (Santa Cruz Biotechnology, sc-390133), a mouse anti-NLRP3 monoclonal antibody (Santa Cruz Biotechnology, sc-134306), and a mouse anti-NLRP7 monoclonal antibody (Santa Cruz Biotechnology, sc-377190). The primary antibodies were validated and considered to be species cross-reactive by specific binding to native ovine proteins highly expressed in the thymus and lymph nodes. No labeling was present using isotype-matched control antibodies (21, 22) and was suitable for sheep. Horseradish peroxidase (HRP)-conjugated secondary antibodies (1:2,000 for anti-mouse IgG-HRP, BL001A, or anti-rabbit IgG-HRP, BL003A) were used to detect the target proteins. A pro-light HRP chemiluminescence detection reagent (Tiangen Biotech) was used to detect protein signals, and the immunospecific bands were quantified using Quantity One V452 (Bio-Rad Laboratories, Hercules, CA, United States). Meanwhile, housekeeping protein (GAPDH) was detected using an anti-GAPDH antibody (Santa Cruz Biotechnology, Inc., sc-20357, 1:1,000) to normalize the expression levels of the target proteins.

### 2.4 Immunohistochemical analysis

Immunostaining for endometrial tissue was performed according to standard protocols, which involves deparaffinization, rehydration, epitope retrieval, inhibiting endogenous peroxidase activity, and blocking non-specific binding sites (16). Some sections were stained by hematoxylin and eosin. The mouse anti-NOD2 monoclonal antibody (Santa Cruz Biotechnology, sc-56168) or the mouse anti-CIITA monoclonal antibody (Santa Cruz Biotechnology, sc-13556) was diluted at a 1:200 ratio. For the negative controls, the primary antibodies were replaced with antiserum-specific isotypes at the same protein concentration. An appropriate HRP-conjugated secondary antibody was used (1:500 for anti-mouse IgG-HRP, BL001A), and nuclei were stained with hematoxylin. The immunostaining signals were detected using a DAB kit (Tiangen Biotech) and imaged using a light microscope (Nikon Eclipse E800, Japan) with a digital camera (DP12). The data analysis was performed by independently assigning an immunoreactive intensity score ranging from 0 to 3 to the images by four observers, as described previously (23).

### 2.5 Statistical analysis

The data for relative expression levels of NLRs were analyzed as a completely randomized design with six animals per group using the Proc Mixed models of SAS (Version 9.1; SAS Institute, Cary, NC). For endometria from different stages of gestation or pregnancy status, the model included random effects for each ewe and fixed effects for the stage of gestation, pregnancy status, and the interaction between the stage of gestation and pregnancy status. The relative expression levels of different groups were compared using the Duncan method, with the experiment-wise type ± error controlled at 0.05. Data are presented as least squares means. The Kruskal–Wallis test was used for analyzing the immunohistochemical data. A *p*-value of <0.05 was considered statistically significant.

## 3 Results

### 3.1 Relative expression levels of *NOD1, NOD2, CIITA, NAIP, NLRP1, NLRP3,* and *NLRP7* mRNA in the endometrium

**Figure 1:**
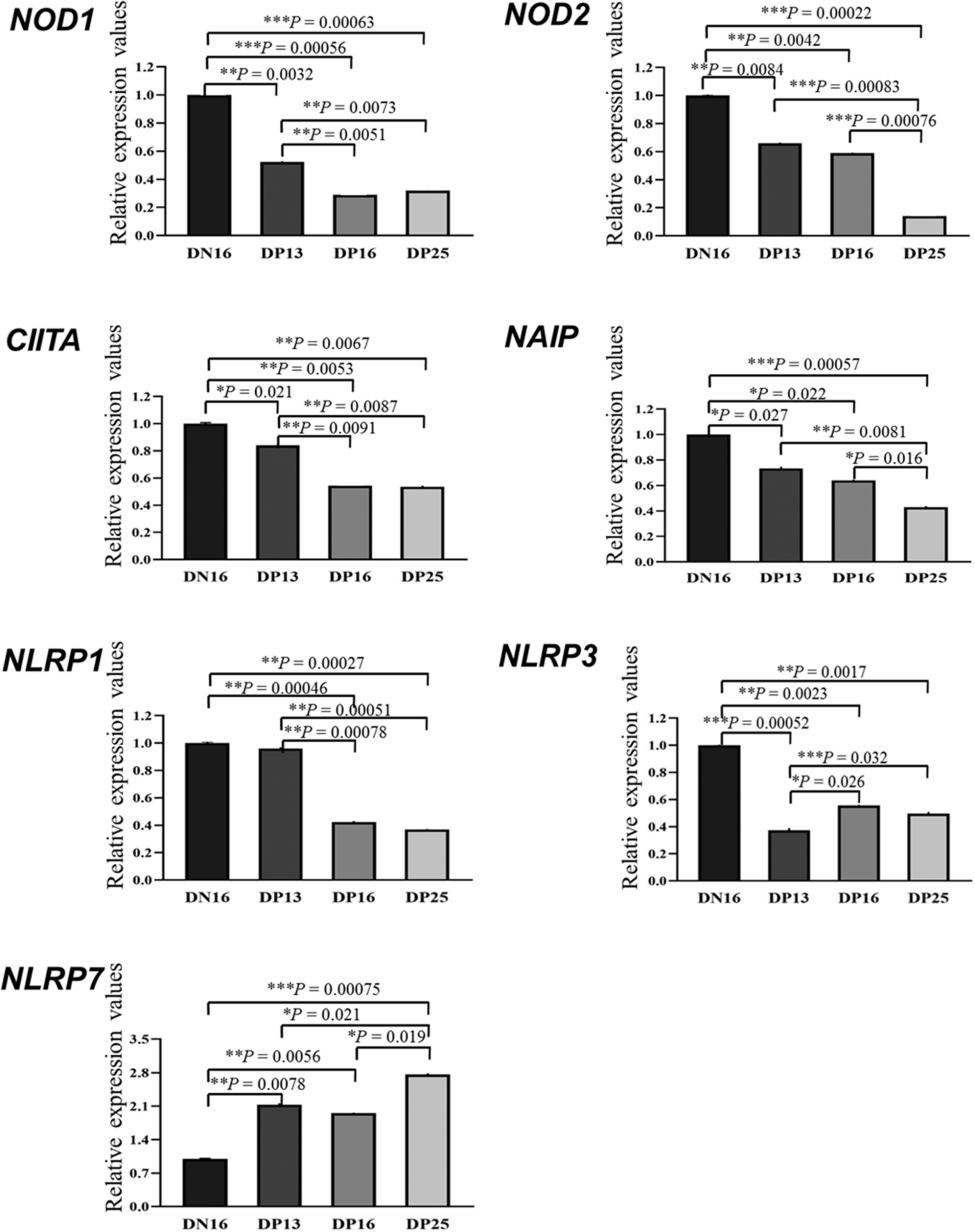
Relative expression values of the nucleotide-binding domain (NOD)-like receptor (NLR) mRNA in the endometrium measured by quantitative real-time PCR in ewes. The NLR family mainly includes NOD1, NOD2, class II histocompatibility complex (MHC) transactivator (CIITA), neuronal apoptosis inhibitor protein (NAIP), nucleotide-binding oligomerization domain, leucine-rich repeat, and pyrin domain-containing (NLRP). The relative levels of mRNA are calculated using the reference gene (*GAPDH*) and day 16 of non-pregnancy as normalization controls. DN16 = Day 16 of non-pregnancy; DP13 = Day 13 of pregnancy; DP16 = Day 16 of pregnancy; DP25 = Day 25 of pregnancy. *p*-values between 0.01 and 0.05 are shown with one (*) asterisk, *p*-values between 0.001 and 0.01 are shown with two (**) asterisks, and p-values less than 0.001 are designated with three (***) asterisks.

It is shown in Figure 1 that there were decreases in mRNA values of *NOD1* and *CIITA* during early pregnancy compared to the non-pregnant ewes (*p* < 0.01), and the values of *NOD1* and *CIITA* at day 13 of pregnancy were higher than those at days 16 and 25 of pregnancy (*p* < 0.01). In addition, early pregnancy inhibited the mRNA expression of *NOD2* and *NAIP*, and the values of *NOD2* and *NAIP* at day 25 of pregnancy were lower than those at days 13 and 16 and of pregnancy (*p* < 0.05). Furthermore, mRNA values of *NLRP1* were higher at day 16 of the estrous cycle and day 13 of pregnancy than at days 16 and 25 of pregnancy (*p* < 0.01). Moreover, mRNA values of *NLRP3* during early pregnancy were lower compared to the non-pregnant ewes (*p* < 0.01), and the value of *NLRP3* at day 13 of pregnancy was lower than that at days 16 and 25 and of pregnancy (*p* < 0.05). However, the expression value of *NLRP7* mRNA was the lowest at day 16 of the estrous cycle among the four groups, and the value of *NLRP7* at days 13 and 16 of pregnancy was lower than that at day 25 and of pregnancy (*p* < 0.05).

### 3.2 Expression of NOD1, NOD2, CIITA, NAIP, NLRP1, NLRP3, and NLRP7 proteins in the endometrium

**Figure 2:**
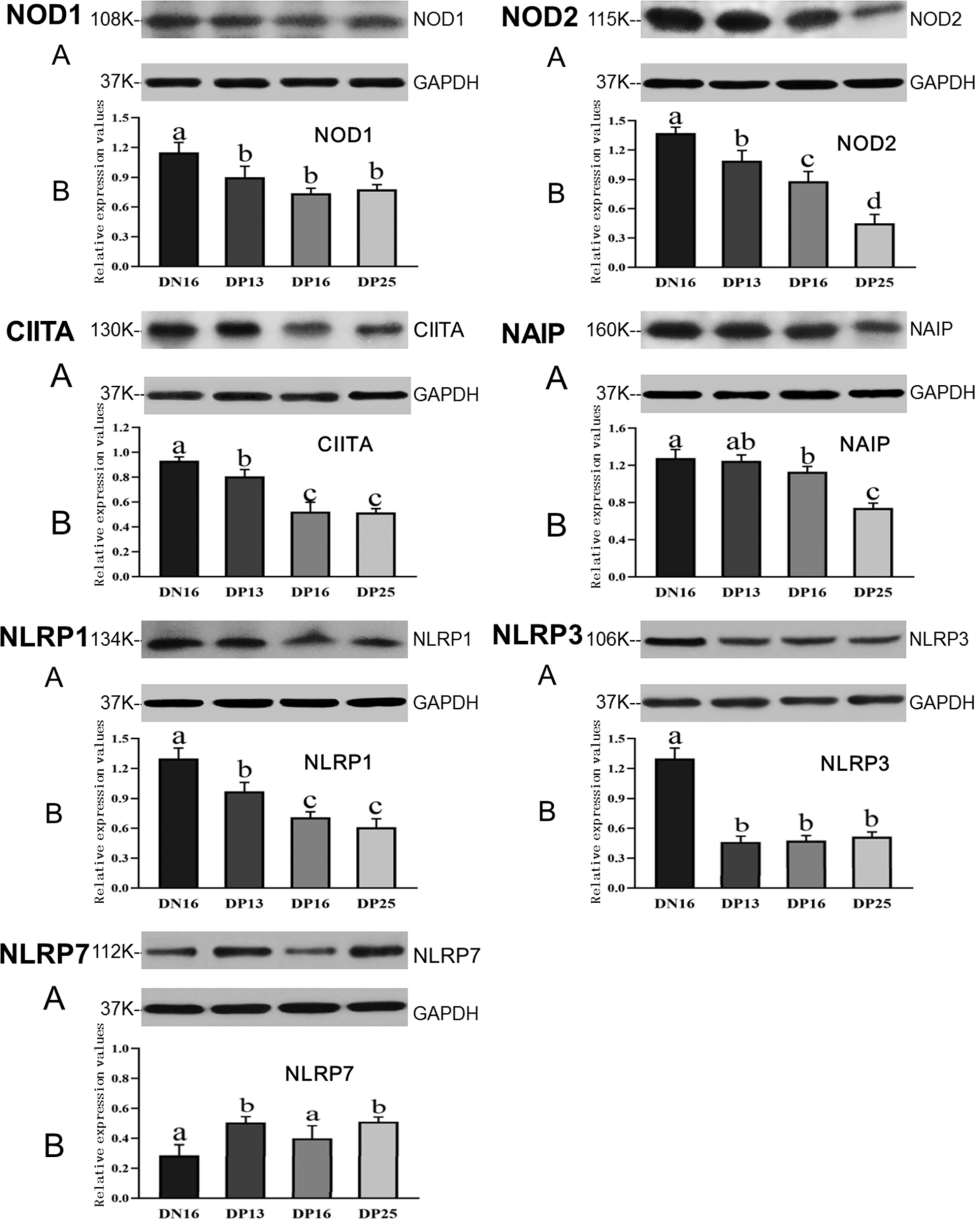
Expression of NOD1, NOD2, CIITA, NAIP, NLRP1, NLRP3, and NLRP7 proteins in the endometrium analyzed by the Western blot in ewes. A: Representation of the Western blot; B: Data of the Western blot; DN16 = Day 16 of non-pregnancy; DP13 = Day 13 of pregnancy; DP16 = Day 16 of pregnancy; DP25 = Day 25 of pregnancy. Significant differences (*p* < 0.05) are indicated by different letters.

Figure 2 shows that the expression of NOD1 and NLRP3 proteins is suppressed in early pregnancy compared to day 16 of the estrous cycle (*p* < 0.05), and there was no significant difference among the pregnant ewes in the expression of NOD1 and NLRP3 proteins (*p* > 0.05). The NOD2 protein was downregulated during early pregnancy, and there was a decreasing expression of the NOD2 protein from day 13 to day 25 of pregnancy (*p* < 0.05). Expression levels of CIITA and NLRP1 proteins during early pregnancy were lower compared with that at day 16 of the estrous cycle (*p* < 0.05), and the expression levels were higher at day 13 of pregnancy than at days 16 and 25 of pregnancy (*p* < 0.05). In addition, expression levels of NAIP protein were higher at day 16 of the estrous cycle than at days 16 and 25 of pregnancy (*p* < 0.05), and the expression value of NAIP at day 25 of pregnancy was the lowest among the four groups (*p* < 0.05). However, expression levels of NLRP7 were lower at day 16 of the estrous cycle and day 16 of pregnancy compared to days 13 and 25 of pregnancy (*p* < 0.05) (Figure 2).

### 3.3 Immunohistochemistry for NOD2 and CIITA proteins in the endometrium

**Figure 3:**
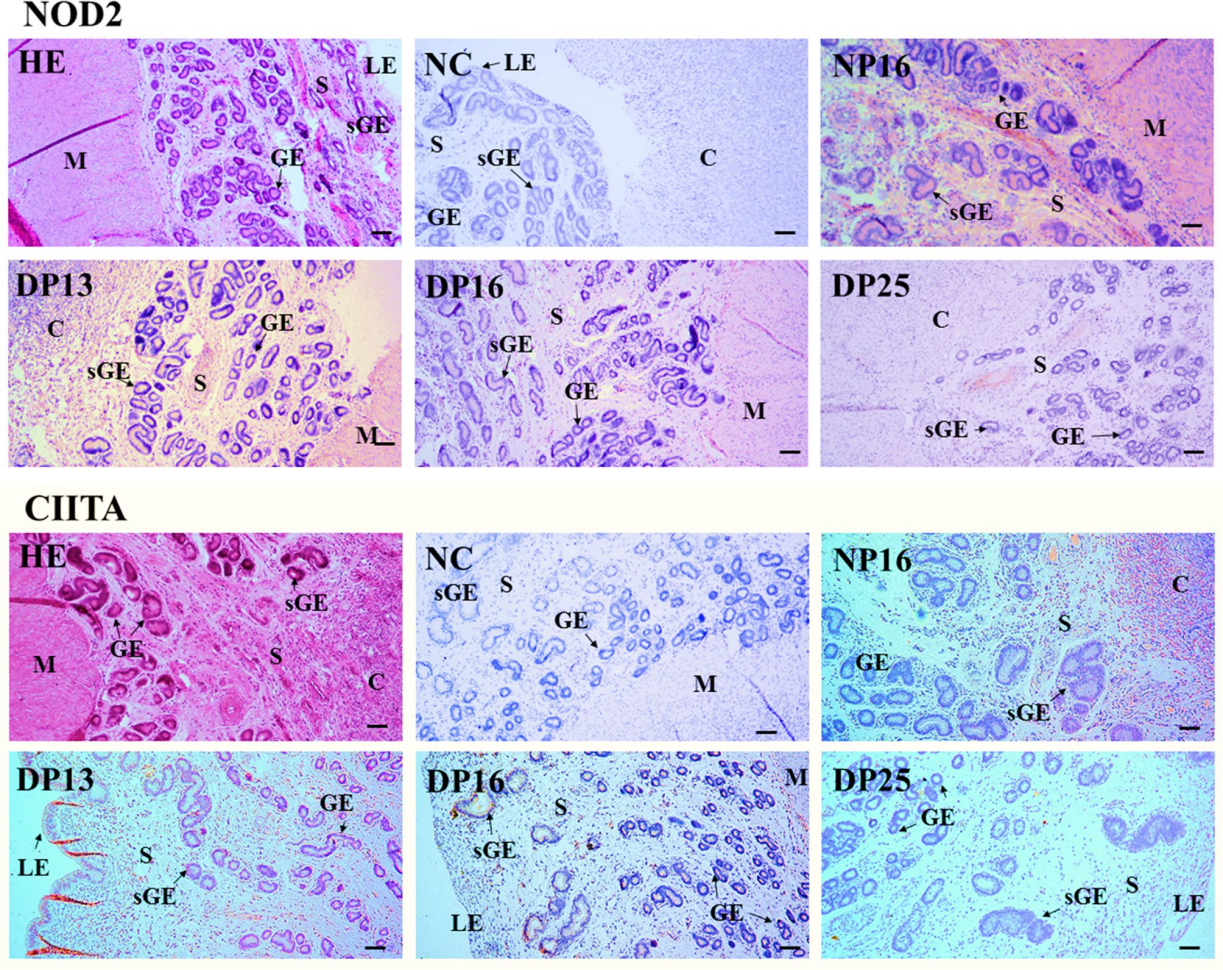
Immunohistochemical localization of NOD2 and CIITA proteins in the endometrium. The NOD2 protein was mainly located in the stroma (S), caruncle (C), and myometrium (M), but the CIITA protein was mainly limited to the luminal epithelium (LE), superficial glandular epithelium (sGE), caruncle (C), and stroma (S). The staining intensities for NOD2 protein were stronger, strong, weak and weak, while that for CIITA protein were strong, stronger, strong and weak for the endometria from day 16 of the estrous cycle, and endometria from days 13, 16, and 25 of pregnancy, respectively. HE, stained by hematoxylin and eosin; NC, negative control; GE, glandular epithelium; DN16, Day 16 of non-pregnancy; DP13, Day 13 of pregnancy; DP16, Day 16 of pregnancy; DP25, Day 25 of pregnancy. Bar = 100 μm.

The NOD2 protein was found mainly in the stroma, caruncle, and myometrium, but the CIITA protein was limited largely to the luminal epithelium, superficial glandular epithelium, caruncle, and stroma (Figure 3). The staining intensities for the NOD2 protein were 0, 3, 2, 1, and 1, for the negative control, endometria from day 16 of the estrous cycle, and the endometria from days 13, 16, and 25 of pregnancy, respectively. Similarly, the staining intensities for the CIITA protein were 0, 2, 3, 2, and 1, following the same order (Figure 3). The scale for staining intensity is as follows: 0 = negative; 1 = weak; 2 = strong; 3 = stronger.

## 4 Discussion

NOD1 plays key roles in regulating both the innate immunity and adaptive immunity by recognizing and binding endogenous to damage-associated molecular patterns, which leads to the activation of NF-κB and mitogen-activated protein kinase signaling pathways (24). Placental tissues and trophoblasts express NOD1, but NOD1 agonists stimulate a proinflammatory cytokine profile, which triggers preterm delivery in mice (25). There is a significant upregulation of NOD1 and inflammatory cytokines in the ectopic endometrium, suggesting that NOD1 is associated with endometriosis (18). The transcription of *NOD1* in the endometrium declines from the first, to the third trimester of pregnancy in cows (19). It has been reported that the concentrations of P_4_ in plasma are higher during early pregnancy than on day 16 of the ovine estrous cycle (26). In this study, *NOD1* mRNA and protein levels were downregulated in the endometrium during early pregnancy, which may attenuate uterine inflammatory responses and be modulated by IFNT from the conceptus and P_4_.

NOD2 can maintain immune homeostasis by activating the proinflammatory transcriptional program, autophagy, and endoplasmic reticulum stress; however, a high level of NOD2 is related to several inflammatory diseases (27). NOD2 enhances the expression of prolabor mediators in human fetal membranes and myometrium by regulating NF-κB activation and transcription (28). The expression level of *NOD2* mRNA was higher in the chorioamniotic membranes of women with spontaneous preterm labor and acute histologic chorioamnionitis compared to those without acute histologic chorioamnionitis (29). Our results showed that *NOD2* mRNA and protein levels declined in the endometrial epithelium during early pregnancy, and NOD2 protein was mainly located in the stroma, caruncle, and myometrium. Therefore, the downregulation of NOD2 may be helpful for embryo implantation and related to early pregnancy signals and high levels of P_4_.

CIITA is a master transactivator of MHC class II genes, and MHC class II proteins play essential roles in immune responses (30). There is a suppression of MHC class II molecule expression in fetal trophoblasts, which is associated with specific inhibition of the CIITA gene (31). CIITA reduces type 2 helper (Th2)-type cytokine expression but has no effects on Th1 differentiation (32). There is a Th2-dominant cytokine pattern during normal human pregnancy, which is mediated by P_4_ (33). Ovine placentation has a limited requirement for local immunoregulation compared to the placentation of primates and rodents (34); however, the patterns of T helper/Treg cells in the placentae have effects on ovine pregnancy outcomes (35). It has been reported that antigen-specific peripheral immune responses are unaltered during normal pregnancy in sheep. However, further studies on maternal–fetal interactions and immune regulation during pregnancy are needed (36). In this study, the expression level of CIITA in the endometrium was lower during early pregnancy compared to non-pregnancy, and the CIITA protein was limited mainly to the luminal epithelium, superficial glandular epithelium, caruncle, and stroma. Thus, the downregulation of CIITA may be associated with a high level of P_4_ and be beneficial for pregnancy establishment.

NAIP proteins are essential for the innate immune sense, and interferon regulatory factor 8 modulates their gene expression (37). Expression of the NAIP protein is higher in the first trimester placentas than the term placentas, and the NAIP protein is limited to the cytoplasm of villous cytotrophoblast cells, syncytiotrophoblast, villous mesenchymal cells, and villous endothelial cells in humans (38). In addition, induced endometrial inflammation alters histotrophic composition and reduces conceptus growth, which increases pregnancy loss in cows (39). Altered endometrial immune gene expression has detrimental effects on embryo survival on day 7 after estrus in beef heifers (40). It was found in this research that the expression level of *NAIP* mRNA and protein gradually decreased during pregnancy. Thus, the downregulation of NAIP may attenuate the innate immune sense to decrease the immune responses and be necessary for achieving endometrial immune tolerance and pregnancy establishment.

NLRP1 inflammasome converts pro-caspase-1 into caspase-1 by autocleavage after stimulation, which results in the secretion of innate proinflammatory cytokines, including IL-1β and IL-18, to contribute to innate immunity (41). Preeclampsia is the main cause of high maternal and fetal morbidity and mortality in humans, and there is higher endogenous activation of NLRP1/NLRP3 inflammasomes and protein expression of IL-1β, IL-18, and tumor necrosis factor-α in the peripheral blood monocytes of preeclamptic pregnant women compared to normal controls (42). Oxidative stress decreases expression levels of glutathione and glutathione peroxidase 4 and increases the expression of the NLRP1 inflammasome in both the rat model and the placental trophoblast cell model, which is implicated in severe pregnancy complications (43). Our data showed that early pregnancy inhibited the expression of *NLRP1* mRNA and protein in the endometrium. Thus, the downregulation of NLRP1 may modulate uterine innate immune responses and be necessary for a successful pregnancy.

Stress-driven inflammation results in organelle dysfunction, which is activated by the NLRP3-inflammasome complex (44). There is a high level of NLRP3 expression in granulosa cells from patients with ovarian insufficiency, and the NLRP3 inflammasome has negative effects on female fertility (45). Activating the NLRP3 inflammasome disrupts the inflammatory microenvironment in the uterus, which leads to excessive inflammation at the maternal–fetal interface in rat preeclampsia (46). The NLRP3 inflammasome is activated in the myometrium tissues and amnion and chorion deciduas, which are associated with labor onset at term and preterm in humans and mice (47). Our results revealed that the expression level of NLRP3 was downregulated during early pregnancy. Thus, the downregulation of NLRP3 may be involved in pregnancy establishment and is regulated by IFNT and P_4_ in ewes.

As a sensor for the inflammasome, NLRP7 has effects on endometrial remodeling and placental development, which play key roles during early pregnancy (48). Expression of *NLRP7* is abundant in decidual macrophages during the first trimester of pregnancy. These macrophages are involved in decidualization and macrophage differentiation to maintain endometrial hemostasis and reproductive success in humans (49). NLRP7 plays key roles in the acquisition of immune tolerance in trophoblast cells by regulating key immune tolerance-associated factors, improving trophoblast proliferation, and decreasing its differentiation during normal pregnancy (50). In this study, the *NLRP7* mRNA and protein increased during early pregnancy. Thus, the upregulation of NLRP7 during early pregnancy may contribute to endometrial remodeling and embryo implantation and be modulated by the early pregnancy signal and P_4_.

**Figure 4:**
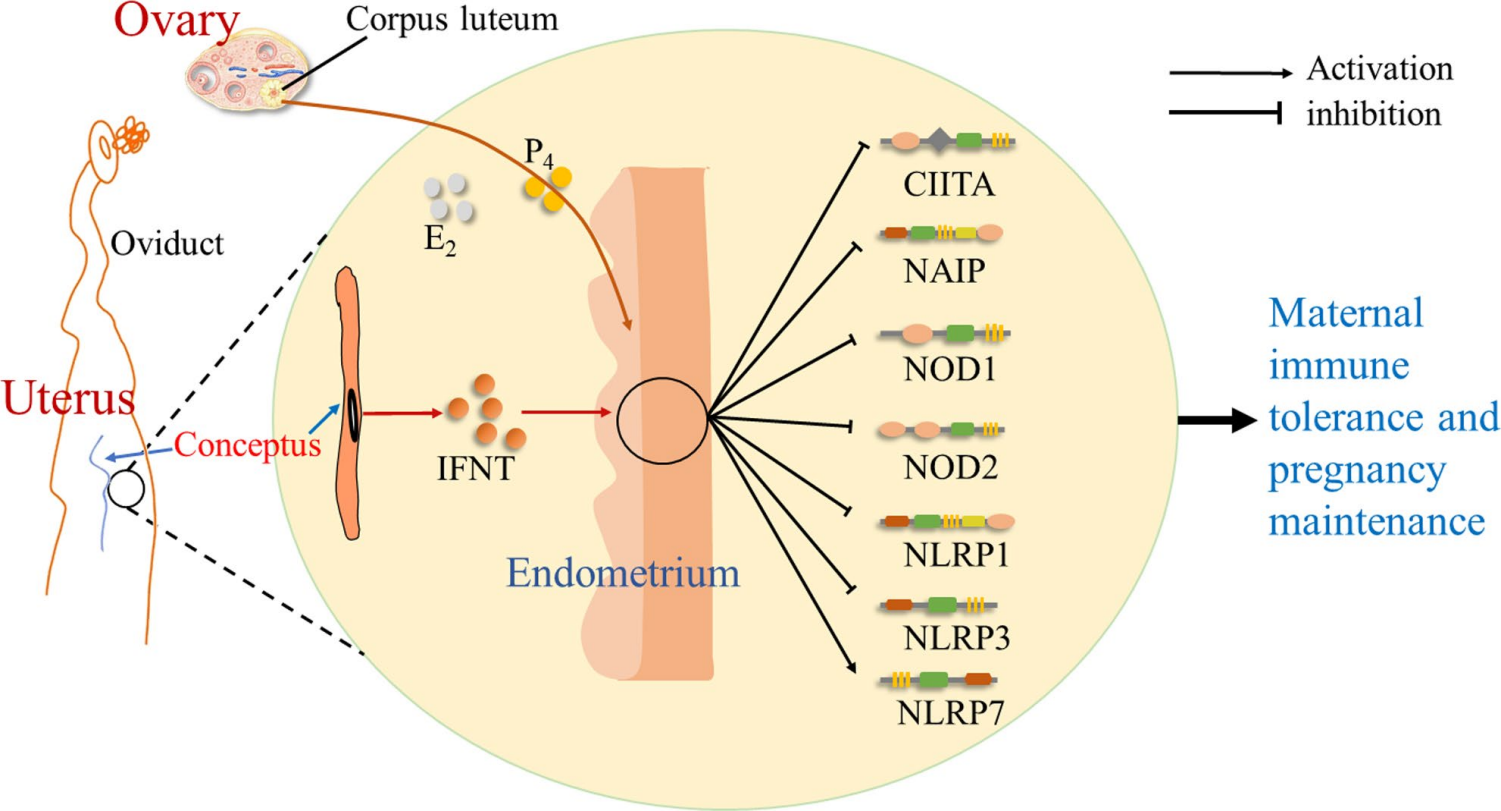
Sketch of NOD-like receptors in the endometrium during early pregnancy in sheep. It is suggested in this study that early pregnancy signal (IFNT) from the conceptus, as well as progesterone (P_4_) and estradiol (E_2_) from the ovary, exerts effects on the endometrium, which change the expression of NOD-like receptors, including CIITA, NAIP, NOD1, NOD2, NLRP1, NLRP3, and NLRP7, and modulates endometrial immune responses to contribute to maternal immune tolerance and pregnancy establishment.

It is suggested that during early pregnancy in sheep, early pregnancy signal (IFNT) from the conceptus, as well as P_4_ and estradiol (E_2_) from the ovary, exerts effects on the endometrium, which modulate the expression of NLRs. There is an upregulation of NLRP7 expression, but the expression of CIITA, NAIP, NOD1, NOD2, NLRP1, and NLRP3 is downregulated during early pregnancy. Thus, during early pregnancy, pregnancy signal (IFNT), together with a high level of P_4_ and a low concentration of E_2_, change the expression of NLRs in the endometrium, which modulates endometrial immune responses and contributes to pregnancy establishment (Figure 4).

## 5 Conclusion

Early pregnancy inhibited the expression of NOD1, NOD2, NAIP, CIITA, NLRP1, and NLRP3 in the endometrium, but the expression level of NLRP7 upregulated during early pregnancy. In addition, NOD2 and CIITA proteins were located in the endometrium in a protein type-, cell type-, and pregnancy status-specific manner. Therefore, early pregnancy changed the expression of the NLR family in the ovine endometrium, which may be involved in the regulation of endometrial immune tolerance and is regulated by IFNT and P_4_ in sheep.

## Data availability statement

The raw data supporting the conclusions of this article will be made available by the authors, without undue reservation.

## Ethics statement

The animal study was approved by the Hebei University of Engineering Animal Care and Use Committee (application number 2019-017). The study was conducted in accordance with the local legislation and institutional requirements.

## Author contributions

LZ: Investigation, Methodology, Writing – original draft, Writing – review & editing. JC: Investigation, Methodology, Writing – original draft, Writing – review & editing. XW: Investigation, Methodology, Writing – original draft, Writing – review & editing. ZY: Validation, Writing – original draft, Writing – review & editing. HD: Formal analysis, Writing – original draft, Writing – review & editing. LY: Conceptualization, Project administration, Supervision, Writing – original draft, Writing – review & editing.
